# Triple-P e-Parenting to Improve Awareness of Psychiatric Nurses on Preventing Cyberbullying in Adolescents

**DOI:** 10.3390/healthcare11010019

**Published:** 2022-12-21

**Authors:** Iyus Yosep, Iqbal Pramukti, Hana Rizmadewi Agustina, Kurniawan Kurniawan, Habsyah Saparidah Agustina, Rohman Hikmat

**Affiliations:** 1 Department of Mental Health, Faculty of Nursing, Universitas Padjadjaran, Bandung 40132, Indonesia; 2 Department of Community Nursing, Faculty of Nursing, Universitas Padjadjaran, Bandung 40132, Indonesia; 3 Department of Fundamental Nursing, Faculty of Nursing, Universitas Padjadjaran, Bandung 40132, Indonesia; 4 Department of Nursing, Politeknik Negeri Subang, Subang 41211, Indonesia; 5 Faculty of Nursing, Universitas Padjadjaran, Bandung 40132, Indonesia

**Keywords:** cyberbullying, psychiatric nurses, seminar, Triple-P e-Parenting

## Abstract

The impact of cyberbullying includes mental health problems and an increased risk of suicide. Psychiatric nurses play an important role in reducing the impact of cyberbullying on students. Nurses, educators, and counselors also play a role to prevent cyberbullying by improving awareness. The purpose of this study is to explore the awareness and involvement of mental nurses and their motivation and confidence in preventing cyberbullying. The research method used in this study was a quasi-experimental pre-post tests study. The sample in this study included 192 respondents. Data analysis used a *t*-test (pair *t*-test). This study shows that seminars about cyberbullying and Triple-P e-Parenting are effective in increasing nurses’ awareness regarding the incidence of cyberbullying in adolescents (*p* < 0.001). The aspects that influence the increase in awareness of mental nurses are attitude (*p* < 0.001) and parenting (*p* < 0.001). Awareness of nurses as parents related to cyberbullying is important to prevent cyberbullying in adolescents. Seminars about cyberbullying, the role of parents, and how to prevent cyberbullying are effective in preventing cyberbullying in adolescents by paying attention to the experiences of parents who have children affected by cyberbullying.

## 1. Introduction

Cyberbullying is a negative action taken, such as sending text messages, photos, meme images, and videos, to someone’s social media account with the aim of satirizing, insulting, harassing, discriminating and even persecuting individuals [1]. Cyberbullying causes psychological harm, pain, suffering, and is proven to have a traumatic impact on victims [2]. Victims of cyberbullying are individuals who are targeted by perpetrators bullying on social media [3]. The forms of cyberbullying that are accepted by cyberbullying victims included having been ignored, disrespected, threatened, and rumors spread by other people [4].

In Indonesia, the prevalence of adolescent victims of cyberbullying is reported to be 80%, and almost every day teenagers experience cyberbullying [5]. According to the United Nations Children’s Fund (UNICEF), the proportion of cyberbullying victims in Indonesia reached 41–50% [6]. The Indonesian Child Protection Commission stated that among students in schools who became victims of cyberbullying, there were 83 victims of bullying on social media, with 32 boys and 51 girls [7].

Cyberbullying experiences can have a significant impact on a adolescent’s emotional and psychological well-being [8]. The mental health condition induced by cyberbullying victims is negative affect (psychological distress), e.g., anxiety, emotional stress, depressive symptoms, suicidal ideation and attempts [9]. Another study showed that negative effects of cyberbullying include frustration, anxiety, depression, fatigue, feeling reduced self-esteem, difficulty concentrating, moody, self-blame, irritability, and suicide [10]. The negative effects of cyberbullying are feelings of sadness, hopelessness, and helplessness [11,12]. Other studies have shown that cyberbullying causes sadness, anger, frustration, shame, or fear [9,13].

Nurses are health workers who have a role in preventing and reducing the incidence of cyberbullying. The role of nurses as educators can provide education to the public related to prevention and programs to overcome cyberbullying [14]. One program to prevent cyberbullying is e-parenting. E-parenting is a model of parenting that is adapted to the habits of children who are so familiar with digital devices [15]. A positive parenting program (Triple-P) is a form of intervention for families in increasing the knowledge, skills, and confidence of parents. The positive parenting program (Triple-P) includes components of self-sufficiency, parental self-efficacy, self-management, personal agency, and problem-solving. Previous research concluded that Triple-P special care can prevent the impact of cyberbullying in Spain, Europe, and Latin America [16]. Previous studies have shown that parenting can improve the prevention of cyberbullying in children with optimal supervision of children [17,18].

Cyberbullying and e-parenting have been identified as crucial problems among youth in the last decade. The impact of cyberbullying on adolescent are depression, mood disorders, anxiety or attempts to end life [8]. Mental health nurses play dual roles as parents and health workers. Nurses must focus on their role in preventing the impact of cyberbullying on adolescents in hospitals. On the other hand, as parents, they must provide the best parenting for their children [19].

Mental health nurses must have competency in respect of the parents’ awareness and involvement with cyberbullying. Besides of that, nurses have to explore experiences regarding prevention efforts, knowledge, attitude, and self-efficacy for the success of their roles as nurses and as parents [20]. This study aims to explore the awareness and involvement of mental nurses as parents and their motivation and confidence in preventing cyberbullying.

## 2. Materials and Methods

### 2.1. Design

The adopted design in this study was quasi-experimental with pretest-posttest study. Participants consisted of a group that attended a seminar on Cyberbullying & Triple-P e-Parenting. Participants filled out the Parents’ Awareness and Involvement with Cyberbullying instrument before and after the seminar (pre-test and post-test).

### 2.2. Participants

Participants in this study were selected based on data owned by the Cisarua Mental Hospital. The inclusion criteria in this study were nurses who have adolescent children. The sample for this study were 192 nurses who had taken the pre-and post-tests for the webinar activity.

### 2.3. Procedure

Participants are given seminars about the principles of e-positive parenting programs via zoom. Before attending the seminar, participants filled out a pre-test using the Parents’ Awareness and Involvement with Cyberbullying instrument for 15 min. Then, the participants received training on the concept of cyberbullying and the role of the e-parenting program by experts, namely mental nurses and psychologists, which were conducted for 2 × 60 min. This was proceeded with a question-and-answer session for 1 × 30 min. After that, participants filled out the Parents’ Awareness and Involvement with Cyberbullying questionnaire for 15 min.

### 2.4. Data Collection

The instrument used in this research is Parents’ Awareness and Involvement with Cyberbullying, which was sourced from Tal and Prebor in 2020 (translated and adapted for pre-teens by the paper’s author) (1). This questionnaire consists of 6 sub-topics, there are: (a) demographic data of parents; (b) information about children in the last year; (c) experience with children; (d) prevention aspects; attitude; (e) aspects of parental knowledge; self-efficacy; and (f) parenting. This instrument is given to respondents who participate in the webinar until it is finished. The pre-test instrument was filled in before the material activity was completed, and the post-test questionnaire was filled out after the material was completed by the respondent using the Google Form.

### 2.5. Data Analysis

The authors conducted data analysis to find out the effectiveness of the seminar given to mental nurses used a *t*-test (pair *t*-test). The software used in this statistical test is SPSS version 28 statistical software, Geneva, Switzerland.

### 2.6. Ethical Considerations

This study followed the ethical principles approved by the Ethics Committee of The Health Research Ethics Committee Nursing School of PPNI, West Java (No. III/020/KEPK-SLE/STIKEP/PPNI/JABAR/VI/2022). Participants were informed of the study’s objective and advised that they might withdraw out at any time. Nurses Towards Cyberbullying and Triple-P e-Parenting provided written informed permission.

## 3. Results

Respondents in this study consisted of 192 nurses at the Cisarua Mental Hospital from diploma graduates to students of the Master’s in Nursing program. Respondents were given seminars and involved in focus group discussions on cyberbullying and triple p-parenting to prevent and overcome cyberbullying. To consider the effectiveness of the given intervention, the respondents were assessed in the form of pre-test and post-test.

### 3.1. Characteristics of Respondents

This study was conducted on 192 samples of psychiatric nurses at the Cisarua Mental Health Hospital, while the characteristics of the respondents in this study were as follows (Table 1):

Based on the table above, of the 192 respondents who participated in seminars and focus group discussions on cyberbullying and triple p-parenting to overcome cyberbullying, more than half of the respondents were aged 31–50 years (56.3%). Most of the respondents were female (63.5%). Most of the respondents were married (89.6%) and almost half had 2 children (33.3%). More than half of the respondents were nursing diploma graduates, more than half worked in the inpatient ward (acute) (55.2%), and almost half of the respondents had worked in a mental hospital for 11–20 years (39.5%). Almost all respondents had never attended cyberbullying training (93.2%).

### 3.2. Information about Nurses Child in the Last 1 Year

The following is the result of an analysis of the child's habits of psychiatric nurses at the Cisarua Mental Hospital (Table 2).

Based on the table above, from 192 respondents, we obtained data regarding children from nurses one year back. Most children of nurses have a mobile phone with Wi-Fi connection or quota (81.3%) and almost all children of nurses use mobile phones. (90.6%). The activities most carried out by children from nurses were playing online games and playing social media or entertainment, being reported by as many as 81 children.

### 3.3. Effectiveness of Seminar about Cyberbullying & Triple-P e-Parenting

The results of this study on the effectiveness of seminars on cyberbullying and Triple-P e-Parenting are presented in the following table (Table 3):

Based on the table above, the effectiveness of the seminar and FGD towards Cyberbullying & Triple-P e-Parenting can be seen. Seminar and FGD towards Cyberbullying & Triple-P e-Parenting are effective (*p* < 0.005) in preventing and overcoming cyberbullying in 2 aspects, namely attitude (*p* = 0.000) and parenting (*p* = 0.00 *). The results of the study also show that Seminar and FGD towards Cyberbullying & Triple-P e-Parenting are less effective (*p* > 0.005) in improving preventing and overcoming cyberbullying in three aspects, namely preventive aspects (*p* = 0.17), knowledge (*p* = 0.11), and self-efficacy (*p* = 0.11).

## 4. Discussion

This study aimed to obtain an overview of the awareness and involvement of mental health nurses as parents to motivate and increase their confidence in preventing cyberbullying in adolescents.

Based on the results of the characteristics of respondents affected the incidence of cyberbullying, for example, the level of education. A study stated the lower the education of parents, the greater likely they are involved in cyberbullying either as victims or as perpetrators [21]. However, this is contrary with other studies which state that the higher the level of parental education, the greater the possibility of cyberbullying [22]. Victims of cyberbullying are 1.60 times more frequent in adolescents whose educational background is only elementary school compared to high school graduates. Then, cyberbullying is found to be 1.89 times more common in adolescents whose mothers graduated from university than high school. Moreover, children of fathers with high school education experienced cyberbullying 2.10 times more than fathers only have education elementary school [22].

In relation to nurses who have children, 81.3%, having a mobile phone with Wi-Fi which makes it easier for teenagers to access and browse the internet, or using a mobile phone 90.6%. Cyberbullying and cyber-victimization can happen anywhere and internet access via smartphones makes it easier [22,23]. Through mobile phones, cyberbullying perpetrators can easily contact their victims, and hide their identities while sharing insulting, mocking, offensive, inciting, and insulting messages through digital media [24]. In addition, there are internet activities conducted by adolescents in this study, e.g., online games and entertainment, or the use of social media, reported here by as many as 81 people, consistent with several studies which state that parents report that their children use social media, such as Facebook and Instagram [22]. Moreover, 89% of teens have at least one media account social media, and although they used Facebook the most (77.6%) followed by Instagram (72.1%), they also have other social media accounts. Those who use social media are more likely to be victims of cyber and cyberbullying.

Social media has been proven to influence the experiences of cyber-victimization of adolescents aged between 12 and 17 years, where the level of cyber victims and cyber bullies increased [25,26]. Then, a higher frequency of use of information and communication technology causes a higher risk of being a victim. The results of a study on cyber-aggression with a sample of 627 adolescents (12–16 years) in Portugal showed that 63.1% reported being involved in cyber-aggression, with 31.1% claiming to be perpetrator–victim [27]. A previous study showed that the effectiveness of seminars and FGD activities related to knowledge about cyberbullying and triple p-parenting in parents turned out to be very effective in several aspects, e.g., parental attitudes in preventing cyberbullying and the needs of parents for certain parenting patterns to prevent cyberbullying (parenting). This is in line with a study that explains that parental involvement is important in reducing the occurrence of cyberbullying or cyber victimization, the role of parents can be positive by actively supervising and monitoring online activities or by limiting what their children do to significantly reduce the likelihood of their children being abused. children become cyber victims [23].

Meanwhile, the awareness of the need for the development of special parenting patterns for parenting in the effort to prevent cyberbullying is significant. This shows that parents need special strategies, one of which is through the Positive Parenting Program (Triple-P).

Triple-p can facilitate parents understand the importance of parenting in preventing or equipping teenagers with the capacity for self-defense. Research in China on the topic of parenting style and cyber-aggression in Chinese youth reported that parenting style is closely related to aggressive behavior in cyberspace, with a sample of 1796 Chinese students who anonymously filled out questionnaires about parenting style, moral detachment, and moral identity, cyber aggression, and demographic variables [28]. Mediation analysis revealed that parenting style is related to cyber-aggressive behavior through moral education. The parenting style of cyber-aggression is much stronger in adolescents with higher moral identities. This study has practical implications for the importance of parenting by parents and educators on how to anticipate and adapt to the destructive impact of cyber-aggression. A meta-analysis covering all studies that discussed, analyzed, and evaluated the impact of triple positive parenting programs on parents and children, showed that Triple P causes positive changes in abilities related to parenting skills, overcomes child problem behaviors, and improves the well-being of people old [29].

Aspects needed to increase awareness regarding cyberbullying are preventive aspects, knowledge, and self-efficacy. Regarding these aspects, it turns out that parents are not aware of cyberbullying and cyber victimization of their children [22,30]. In a previous study conducted on adolescents, half of the participants stated that their parents had no knowledge of or access to their online activities. This is because parents are less informed about internet use, and they have less control over their online activities [23]. Parents need to be aware of cyberbullying and have adequate knowledge and skills to ensure effective supervision. Education and awareness serve to enhance parents’ ability to engage in meaningful conversations about technology with their children. Since most students surveyed claimed that they would tell their parents if they were bullied online, parents must be sufficiently prepared to engage in these conversations in an appropriate manner.

In addition, on the aspect of self-efficacy of parents based on the results of the study, it turns out that they are not sure they can teach their children in preventing cyberbullying. Self-efficacy is needed to improve self-defense to prevent cyberbullying. Higher self-defense and empathic self-efficacy significantly can increase the frequency of self-defense behavior during cyberbullying [23,31]

Active engagement between parents and their children in safe and productive use of the Internet has an impact on reducing the incidence of cyberbullying [32]. Previous research shows that in preventing cyberbullying, parents must be role models in making good use of social media [33]. This requires good, thoughtful, and considerate action in online interactions.

### Limitation

The limitation of this study is that the sample does not describe the number of nurses in Indonesia. In addition, the short duration of seminar activities can cause the knowledge obtained to be less than optimal. However, this study was able to explore important aspects of preventing cyberbullying in adolescents, namely a significant increase in attitudes and parenting aspects. The results of this study are expected to be used as basic data to develop parenting programs to prevent cyberbullying in adolescents.

## 5. Conclusions

Cyberbullying and Triple-P e-Parenting Seminars and FGDs are very effective in changing attitudes and parenting in dealing with cyberbullying. Psychiatric nurses who have play a role as providers of nursing care as well as parents experienced significant changes in attitude after being given training through seminars involving doctors, mental health nurses, and lecturers as resource persons. The training content focuses on the importance of E-parenting which consists of three main components: (1) to create family friendliness; (2) supportive parental environment, e.g., in mass media, public health care, school work sites, religious organizations, malls, and hospitals; (3) competent parenting, such as, knowledge, skills, and confidence in self-regulation.

## Figures and Tables

**Table 1:** Demographic Characteristics Nurses Towards Cyberbullying & Triple-P e-Parenting (*n* = 192).

Variables	*n* (%)
**Age**	
• 18–30	56 (29.2)
• 31–50	108 (56.3)
• >50	28 (14.6)
**Gender**	
• Male	70 (36.5)
• Female	122 (63.5)
**Marital Status**	
• Married	172 (89.6)
• Single (widow, etc.)	20 (10.4)
**Children Number (total)**	
• 1	60 (31.2)
• 2	74 (38.5)
• >2	58 (30.2)
**Education**	
• Diploma Nursing	119 (62)
• Bachelor	71(37)
• Graduate (Master & Doctoral)	2 (1)
**Longest work place**	
• Outpatient ward	13 (6.8)
• Inpatient ward (Acute)	106 (55.2)
• Inpatient ward (Chronic)	37 (19.3)
• Emergency	8 (4.2)
• Others (children &adolescents, drugs, etc.)	29 (15.1)
**Work duration (years)**	
• 1–5	58 (30.2)
• 6–10	15 (7.8)
• 11–20	76 (39.5)
• >20	45 (23.4)
**Do you have ever follow cyberbullying training?**	
• Yes	13 (6.8)
• No	179 (93.2)

**Table 2:** The Information about nurses child in the last 1 year (*n* = 192).

Variables	*n* (%)
**Does your child have a mobile phone with Wi-Fi connection or quota?**	
Yes	156 (81.3)
No	36 (18.7)
**What tools are used the most your child use for Internet browsing?**	
Computer/Laptop	18 (9.4)
Mobile phone	174 (90.6)
**What activity most your child done on social media? (More than 1 choice)**	
Game online	81 (42.1%)
Finding information	38 (19.8%)
Entertainment	81 (42.1%)
Shopping	19 (9.9%)
Watching film	68 (35.4%)
School assignment	59 (30.7%)
Friends communication	54 (28.1%)
Family communication	41 (42.1%)

**Table 3:** The effectiveness of Seminar about Cyberbullying & Triple-P e-Parenting (*n* = 192).

Outcomes	*t*-Test	Mean ± SD	95% CI	*p*-Value
Children experience	−1.07	−0.04 ± 0.5	−0.11–0.03	0.28
Preventive	−1.36	−0.08 ± 0.78	−0.20–0.03	0.17
Knowledge	−1.6	−0.19 ± 1.57	−0.43–0.04	0.11
Attitude	−3.29	−0.04 ± 1.79	−0.72–(−0.18)	0.000
Self-Efficacy	−1.58	−0.4 ± 3.29	−0.89–0.09	0.11
Parenting	−3.92	−0.591 ± 1.97	−0.89–(−0.29)	0.000

## Data Availability

Not applicable.
